# Developing a Patient Care Co-ordination Centre in Trafford, England: lessons from the International Foundation for Integrated Care (IFIC)/Advancing Quality Alliance integrated care fellowship experience

**DOI:** 10.5334/ijic.2030

**Published:** 2015-05-04

**Authors:** Michael Gregory

**Affiliations:** Trafford CCG, Sale, UK

**Keywords:** patient care co-ordination, AQuA, Fellowship

## Abstract

The NHS and Social Care in England are facing one of the biggest financial challenges for a generation. Commissioners and providers need to work on collaborative schemes to manage the increasing demand on health and social care within a period of financial constraint. Different forms of care co-ordination have been developed at different levels across the world. In the north-west of England, the Trafford health and social care economy have been working through a competitive dialogue process with industry to develop an innovative and dynamic solution to deliver seamless co-ordination for all patients and service users. The strategy is to develop a new Patient Care Co-ordination Centre, which will be responsible for the delivery of co-ordinated, quality care. The Patient Care Co-ordination Centre will work at clinical, service, functional and community levels across multiple providers covering risk stratification, preventative, elective and unscheduled care. I am the clinical lead for the Patient Care Co-ordination Centre and during my year as an Advancing Quality Alliance Integrated Care Fellow, I have had the opportunity to study examples of care coordination from UK and international sites. The learning from these visits has been assimilated into the design process of the Patient Care Co-ordination Centre.

## Introduction

Trafford Clinical Commissioning Group, working in partnership with the social care provider, Trafford Council, is developing a new Patient Care Co-ordination Centre which is an innovative and dynamic solution to deliver seamless care co-ordination for all patients and service users. I am a general medical practitioner, a clinical director at Trafford Clinical Commissioning Group and the clinical lead for the development of the Patient Care Co-ordination Centre.

In 2013, I successfully applied to be an Integration Fellow with the Advancing Quality Alliance. Advancing Quality Alliance is a membership body dedicated to improving health care in the north-west of England. The Integrated Care Fellowship Programme provided an opportunity for people working on system level integrated care to undertake supported personal, professional and organisational learning through a programme of self-directed development. My topic of interest is the co-ordination of elective care delivery and the one-year fellowship programme gave me the opportunity to study, and in some cases visit, leading health and social care economies nationally and internationally.

For my fellowship, I wanted to look further into characteristics of successful approaches to elective care co-ordination and to adapt best practice as a model for the Trafford Patient Care Co-ordination service. I needed to study health care economies which demonstrated:

- Examples of how technology can support consultations between professionals to enable better quality and appropriate referrals;
- Enabling a responsible person to have oversight and accountability between transitions of care between providers;
- Examples of robust use of shared guidelines and referral criteria together with alignment of incentives;
- Use of decision support tools such as care guidelines and protocols;
- An organisational level of focus on continuous quality measurement and improvement.

Being a GP myself, I was also keen to understand systems where primary care played a significant role, unlike many of the models based on supporting complex conditions which tend to involve community health and social care teams working with secondary specialities.

## Background

The Trafford Health and Social Care Economy started its journey towards integration in 2008 with an attempt to create an integrated care organisation [1]. The Trafford experience and the literature suggest that organisational integration alone is unlikely to deliver better outcomes and effort must focus on clinical and service integration [2]. Goodwin (key lessons and markers for integrated care – international foundation for integrated care (IFIC) presentation) took this point further by stating that integration without care co-ordination cannot lead to integrated care. Effective care co-ordination, however, can be achieved without the need for the formal integration of organisations. In the absence of a formal organisational merger, strong care co-ordination needs multiple well-connected provider networks. By its nature, integration is dependent upon the local context, so developments in integration will vary in each different health care and social care economy.

There have been studies on excellent models of care co-ordination in chronic care in the UK and elsewhere. As Goodwin et al. say, “Care co-ordination programmes appear to flourish at the neighbourhood level where the benefits of engagement with local communities sit alongside the need to have close working relationships within multidisciplinary care teams dealing with manageable caseload”. And that, “There is potential to scale up operations through building a number of locality-based approaches to care under the direction of an umbrella organisation. Such an approach might have a bigger impact in improving cost-effectiveness, which otherwise appears to be limited” [3].

Trafford Clinical Commissioning Group has been developing its vision of a Patient Care Co-ordination Centre since the summer of 2013. The Patient Care Co-ordination Centre will work at clinical, service, functional and community levels across multiple providers covering risk stratification, preventative, elective and unscheduled care. The Patient Care Co-ordination Centre will manage unscheduled activity by identifying those at greatest risk of hospital admission and tracking and co-ordinating services to ensure a positive experience of the health and social care system. In addition, it will monitor outpatient referrals, align appointment times to a person's availability and transport needs, avoid duplication of investigations and reduce unnecessary appointments.

**Figure 1.**
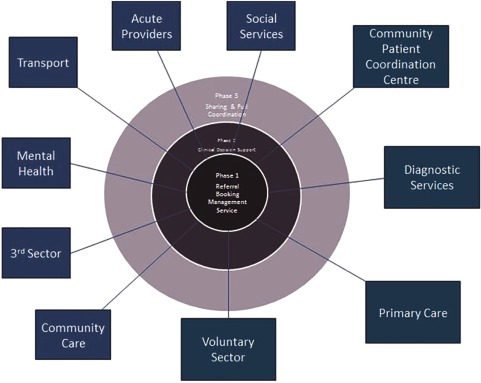
Outline model for Patient Care Co-ordination Centre

The procurement of the Patient Care Co-ordination Centre has been through a competitive dialogue process with bidders over a period of 15 months and has included face-to-face sessions covering functional and technological clarification, patient experience and engagement, service management and deployment. The service is planned to go live from September 2015 (Figure 1).

The Patient Care Co-ordination Centre aligns with the Trafford strategic direction for integrated services and is consistent with national policy. The Better Care Fund, announced soon after the development work began, created a pooled budget to enable health and social care to work together to improve outcomes for people. Clinical commissioning groups can use the funding to ensure that health and care support work together by sharing data and improving continuity of care, acting earlier so that people can stay healthy and independent at home, and delivering care that is centred on individual needs. The Patient Care Co-ordination Centre also fits with the Five Year Forward View, announced in October 2014, which supported a move towards innovative models of care to suit local needs.

“Care co-ordination” has no standard definition but is a term that is used interchangeably with “integrated care”, “case management” and multidisciplinary care” (Goodwin). Care co-ordination can be perceived at a process or team level:

A person-centred, assessment-based, interdisciplinary approach to integrated health care services in a cost-effective manner in which an individual's needs are assessed, a comprehensive care plan developed, and services managed and monitored by an evidence based process usually involving named care co-ordinators. [4]

This model of integration is seen in the management of the complex patient and there are many examples in the literature of a team-based approach of care planning to reduce unscheduled admissions for populations of people with chronic or complex conditions [5, 6]. Care co-ordination, therefore, is seen as particularly relevant to patients with chronic and complex medical conditions and this would be justified from the potential cost-savings and improved outcomes.

However, many people pass through the health care system on an elective basis and this is a potential area that would benefit from better co-ordination of care. For elective care, we know that clinical information can be missing during hand overs, medication errors occur due to a lack of medicines reconciliation between providers, adverse events can occur due of lack of information and that there can be duplication of investigations. Often patients make the connections between services for themselves, but a system can add value by connecting providers for them [7].

Therefore, for my fellowship, I wanted to look at successful approaches to elective care co-ordination which focused on improving transitions from primary care to other parts of the health and social care system.

## Case studies

During my career, I have been fortunate to visit organisations that are exemplars of integration such as the Veterans Administration and Kaiser Permanente in the USA and Jonkoping in Sweden. These health care economies have many of the characteristics that support care co-ordination at clinical, system and organisational levels. Many of the successful elements of these establishments are not currently transferable to my locality.

I have had also the opportunity to visit Intermountain Health care in Utah, USA and, during my fellowship year, Canterbury District Health Board in New Zealand. I believe that these two systems do demonstrate innovative systems for elective care co-ordination with principles and processes that could be applied to an English health care economy.

### Intermountain health care

Intermountain Health care, located in Utah and South-eastern Idaho, is a not for profit vertically integrated system of 22 hospitals, a medical group with more than 185 physician clinics, and an affiliated health insurance company. In the early part of the twenty-first century, it was regularly ranked as one of the best integrated systems in the USA. Intermountain has also received several other awards for the organization's pioneering use of electronic medical records and evidence-based medical care guidelines.

In 1986, Intermountain set up an Institute for Health Care Delivery Research. The aim of the Institute was, amongst other things, to provide leadership in recommending strategic priorities regarding health care delivery and to provide data and statistical services and training in quality improvement. A quality improvement training programme, the advanced training programme in health care delivery improvement was established in 1992 which offered clinicians and non-clinicians training in improvement methodology. A shorter mini advanced training programme and a two day course in quality improvement have been added to the course offerings.

Since 1988, Intermountain Health care has applied to health care delivery the insights of W. Edwards Deming's process management theory, which says that the best way to reduce costs is to improve quality. Intermountain achieved such quality-based savings through measuring, understanding and managing variation among clinicians in providing care. Intermountain created data systems and management structures that increased accountability, drove improvement and produced savings.

Intermountain conducted an analysis of the clinical processes that comprised the bulk of care provided by their facilities. The prioritization criteria included patient volumes, cost per case (inpatient and outpatient), variation in clinical quality, team-based processes of care and social equity. As a result of these, nine programmes were identified as priorities. These have been grouped into eight “Clinical Programmes” around clinical areas, such as cardiovascular medicine or women and newborn, and one programme for Primary Care. Within these programmes, multidisciplinary clinical guidance teams devise and review agreed pathways for a particular care delivery process. Examples in the Primary Care Programme include asthma and diabetes, weight management and anticoagulation management.

In these care pathways, clinicians take responsibility for bringing about improvements through developing guidelines, measurement and peer review. This work is supported by real-time information systems and decision support tools which enable clinicians to follow the pathways. However, the organisational culture is to permit clinicians to deviate from a pathway if that is in the interests of the patient. Then, by relying on good outcome data, pathways can be amended if a different route on a pathway produces a better set of outcomes.

Brent James, the Executive Director for the Institute has argued that the variation seen in medical practice is due to the concept of “clinical uncertainty” as a result of the complexity of clinical practice, a lack of valid information identifying best care across a range of choices, and physicians’ reliance upon subjective recall in making clinical judgments [8]. This type of support system, by having standardised pathways, documents and information, liberates clinicians’ minds to concentrate on the patient's clinical problem and gives them freedom to vary from a pathway based on their professional judgement and the patient's needs.

### Canterbury health board

The New Zealand Health and Social care system is made up of 20 Health Boards funded through central government. One of them, Canterbury District Health Board, spends NZ$1.45bn on its population of around 510,000. Christchurch is the main city within the Health Board with a population of around 400,000 people. There are some 130 general practices in its area, with most GPs being part of a GP provider organisation called Pegasus Health.

In 2006, the system was in crisis due to escalating waiting list times for hospital outpatient appointments. After a number of warnings to Health Boards, the Ministry removed 5000 people from the waiting list and referred them back to primary care.

As a result, a group of clinicians reviewed the 5000 patients. A third of the patients were appropriately referred back into the hospital system. Another third were managed in other ways – being sent back to the GP with advice, or had further investigations, or went to allied health professions. The remaining third really did not need to be seen by the hospital at all. There was a question that the GP had which could easily be answered if they had access to the appropriate information. Perversely, the poorer quality referrals were seen sooner because the consultants could not be sure how safely the patient could wait to be seen.

This crisis spawned the development of Health Pathways in 2008. The pathways are local agreements on best practice, created and are maintained by bringing together hospital doctors and GPs to agree a patient pathway for a particular condition. They spell out which treatments can be managed in the community and what tests GPs should carry out before a hospital referral. They also include how GPs can access such resources including referral to other GPs whose practices have particular skills.

The main value has been the engagement that sits underneath the pathways, encouraging collective and distributed leadership, supporting change over time and making changes sustainable. The process of clinicians sitting together not only resulted in the development of Health Pathways, but also an electronic request management system, and Health Info, a patient-centred website that provides essentially the same information as Health Pathways but in lay language.

There are now more than 500 clinical pathways in total. They are all routinely reviewed at one year, and then once every two years. There is a weekly meeting held by a software organisation called Streamliners were a clinical team answer queries or suggestions about the pathways and make amendments. Everyone gets a response back so there is real clinical ownership of the pathways. An internal study (in print) has demonstrated that primary and secondary care clinicians have demonstrated a high level of acceptance of the Health Pathways and that it has acted as a change management tool by disseminating information required for successful integration.

The process is facilitated by a proactive group called the Canterbury Initiative, which was set up in 2007 to support primary and secondary care clinicians to facilitate work programmes, ensure implementation of change within agreed timelines and to deliver change. The initiative has produced audit reports of clinical pathways to review utilisation and patient outcomes.

The District Health Board also set up a quality improvement training programme called Xceler8 which trained managers in concepts such a Lean and Six Sigma. There are two further programmes Particip8 and Collabor8, which aims to support teams in developing a change project.

Launched in 2010, the Electronic Referral Request System is an electronic referral system between general practice and other parts of the system. GPs use it to request tests, outpatient referrals, community assessments and specialist advice. For radiological tests, the request is reviewed by a GP with expert knowledge and checked to make sure the correct test request has been made for the patient's condition. This has resulted in a reduction in unnecessary tests.

A distinguishing feature of electronic request management system is that the data go to a central repository. It is a data-rich platform for understanding the demand within the system and for planning future resources, which is yet to be used to its full potential.

There is no incentive paid to the GPs to use the system, they did so because, like the Health Pathways, it made their lives easier. The system is installed on the GP's desktop and referral request forms are pre-populated from the GP's clinical system with information that includes the patient's condition aligned to the criteria in the Health Pathway. There is a one click link to Health Pathway as well.

All links between the electronic systems are one click, with no extra passwords being added. This was felt to be important because asking GPs to have to use additional log in information would reduce engagement. According to Carolyn Gullery, General Manager for Planning & Funding at Canterbury District Health Board, the aim is to make things “better on Monday” – to improve the efficiency and effectiveness of the health system by providing tools and improving processes for the delivery of outcomes for patients.

## Reflections from case studies

**Figure 2.**
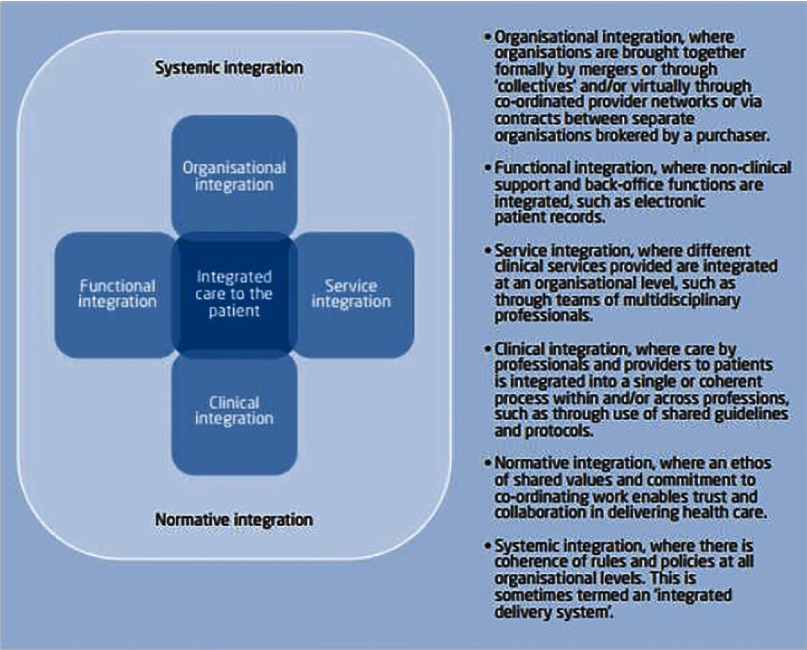
“Fulop's typologies of integrated care” (from [9]) (Source: Adapted from Fulop et al. [2])

Integration as a concept has been defined by typology in terms of organisation, function, service, clinical, normative and systematic (Figure 2).

The two sites, Intermountain and Canterbury share some of these typologies. Intermountain is a vertically integrated organisation with evidence of functional, clinical, normative and systemic integration. Canterbury has teams offering a service level of integration combined with clinical, normative and systemic examples.

They both share a number of common behaviours and models of elective care delivery. Some of these behaviours and models overlap with those of other successful examples of integration I have studied and in the literature as key features for the successful adoption of integrated care (Table 1):

I have chosen Intermountain and Canterbury as case examples as this paper has a focus on elective care co-ordination, but I have noted many these characteristics in other successful integrated systems at Kaiser, the VA and Jonkoping.

Both organisations have developed a sufficient level of business or system intelligence to prioritise clinical processes and pathways. Amongst clinicians there is a culture that values peer review of referrals for feedback and education.

## Incorporating the models in to the proposed Trafford Patient Care Co-Ordination Centre

Trafford Clinical Commissioning Group's vision is for the Patient Care Co-ordination Centre to provide the overarching system which supports the further development of integrated care in Trafford. The centre will operate seven days a week. There will be a single contact number for patients and carers and extended clinical communication between services and health providers, social care and third-sector organisations. All referrals will be monitored to ensure that patients receive timely and appropriate treatment.

While the proposed centre will have the functionality to support anticipatory, elective and unscheduled care across the health and social care economy, this paper reflects on how the models of best practice could be incorporated into the elective care element of the service.

In common with the examples I have described above, in 2009 Trafford had set up “clinical panels” in particular clinical and service areas to allow clinicians from primary and secondary care, community care and patient representatives to develop models of best practice. Trafford also set up a system wide programme based on Intermountain's advanced training programme which trained over 135 people in understanding the tools of quality improvement.

As with Canterbury, the experience from these initiatives was that bringing people together from different parts of the health economy helped form new relationships and a desire to improve health care delivery across the whole system. Following on from the clinical panels, clinicians from primary and secondary care work together in localising prioritised Map of Medicine pathways as standards of best practice. The ability to prioritise pathways has been achieved as a result of an internal business tool that measures activity, cost and variation of outpatient referrals by speciality, source, practice and locality.

For elective care, there has not been an appetite amongst clinicians in Trafford for a referral management system which deflects referrals back to primary care. Instead, a peer review referral programme was set up in areas of high activity and cost. The scheme provides feedback from GPs to GPs on referral quality compared to the Map of Medicine. The scheme has been successful in that reviews in certain clinical pathways improved to a point that it was no longer an efficient use of the system to provide feedback. The review of those pathways has been discontinued and new areas targeted from the list of prioritised pathways identified by business tool information.

The intention is that for elective care, the Patient Care Co-ordination Centre will have a live directory of services and a clinical information system support system. This will include all available referral routes both within the acute, mental health, social care, community care and the voluntary sector. GPs will be able to access localised care pathways which have been designed and developed specifically for Trafford with embedded local service information, local prescribing guidance and patient information. There will be a live Directory of Services integrated electronically as part of the localised care pathways that it is regularly reviewed and maintained. The Directory of Services will provide a catalogue of the services available together with relevant information such as operating times and waiting lists.

Once a referral has been made, clinicians at the centre would ensure appropriate tests have been undertaken and instigate missing tests prior to an outpatient appointment. As with Intermountain and Canterbury, there would be the facility to override a pathway if the referring clinician believes that there is clinical justification. This will be monitored and prompt pathway review or referrer feedback as and when needed.

In a similar manner to Canterbury's Health Pathways, the centre would host a patient information portal providing information about health care and services to enable self-care and to support informed decision making. In a similar way to Canterbury's system, there will be a rich data repository and it is intended that the data captured as a result of the tracking of referrals will support the redesign of services where a gap in service has been identified.

## Summary

The fellowship has given me the opportunity to develop my thinking around care co-ordination. In addition to site visits to Catalonia, Bilbao, London and Christchurch, I was able to attend IFIC conferences in Brussels, Sydney and Wellington and listened to other international leading examples of integrated systems.

The site visits and conference experience has helped me understand that integration is not a single solution that can be transferred across different health economies but is a series of models that are designed to fit local needs. In addition, health care economies are at different stages of the development journey and none have a fixed end point in mind.

There are a number of integrated care providers that demonstrate a set of common characteristics in their management of elective care co-ordination. Many of these are transferable to an English NHS health and social care system and it is intended to incorporate a directory of services, decision support, peer review, agreed pathways and clinical ownership into the development of the care co-ordination centre.

## About the author

Michael is General Practitioner in Altrincham, Cheshire and is the Clinical Director for Strategy and Policy for Trafford Clinical Commissioning Group. He is also a member director of the Advancing Quality Alliance Board and is an Advancing Quality Alliance Integration Fellow for 2014–2015. He is the clinical lead for the development of the Trafford Patient Care Coordination Centre.

Michael's interest is in the improvement of health care delivery and integration. Michael was the Programme Director for a NHS version of Intermountain Health care's world class Advanced Training Programme in Health care Delivery Improvement. The programme was originally set up in Trafford but is now run across the north-west of England by Advancing Quality Alliance as the Advanced Team Training Programme. Michael is the programme director for this course and also lectures on integration and improvement practitioner courses.

Michael continues to remain in front-line clinical practice and this direct experience with patients ensures that he is able to understand the patients’ needs within the health service in service redesign.

## Figures and Tables

**Table 1.**
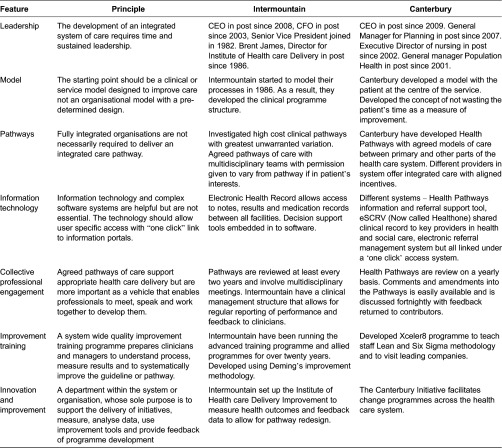
Common behaviours shared by case studies
